# Influence of FOSL1 Inhibition on Vascular Calcification and ROS Generation through Ferroptosis via P53-SLC7A11 Axis

**DOI:** 10.3390/biomedicines11020635

**Published:** 2023-02-20

**Authors:** Sisi Shao, Yaoxin Liu, Wanzi Hong, Yuanxi Mo, Fen Shu, Lei Jiang, Ning Tan

**Affiliations:** 1 Guangdong Provincial Key Laboratory of Coronary Heart Disease Prevention, Department of Cardiology, Guangdong Cardiovascular Institute, Guangdong Provincial People’s Hospital, Guangdong Academy of Medical Sciences, Guangzhou 510080, China; 2 The School of Medicine, South China University of Technology, Guangzhou 510080, China; 3 Guangdong Provincial Geriatrics Institute, Guangdong Provincial People’s Hospital, Guangdong Academy of Medical Sciences, Guangzhou 510080, China

**Keywords:** FOSL1, vascular calcification, ferroptosis

## Abstract

Background: Vascular calcification during aging is highly prevalent in patients with cardiovascular disease; however, there is still no improvement in clarifying the development of vascular calcification. FOSL1 is a transcription regulator belonging to the AP-1 family, which has a unique function in vascular senescence, but its role in vascular calcification needs to be further explored. Methods: Primary mouse vascular smooth muscle cells were isolated and used to construct a calcification model in vitro. Seven-week-old male C57BL/6 mice were used to build the vitD3-induced calcification model in vivo. qRT-PCR and western blot were used to verify the expression of FOSL1 and other genes expressed in vascular smooth muscle cells and aortas. The level of calcification was determined by Alizarin Red S (ARS) staining and the calcium content assay. The level of cellular GSH was detected by the GSH assay kit. Results: Here, we report that FOSL1 was up-regulated after high-calcium/phosphate treatment in both the in vivo and in vitro vascular calcification models. Functional studies have shown that the reduction of FOSL1 attenuates ferroptosis and calcification in vascular smooth muscle cells, as indicated by ARS staining, calcium content assay, and western blot. The inhibition of FOSL1 downregulated the expression of bone-related molecules including Msh Homeobox 2 (MSX2) and tumor necrosis factor receptor superfamily, member 11b/osteoprotegerin (OPG), suggesting that FOSL1 promoted osteogenic differentiation of vascular smooth muscle cells. Furthermore, we found that the ferroptosis-inducing drug erastin can significantly accelerate calcification in the aortic ring while Ferrostatin-1 (fer-1), a drug to protect cells from ferroptosis, can alleviate calcification. Further experiments have shown that inhibiting FOSL1 can promote the expression of ferroptosis-related genes and attenuate calcification. Functionally, cellular GSH levels were increased after the reduction of FOSL1. Conclusions: In this study, we observed a significant protective effect when we reduced the expression of FOSL1 during vascular calcification, and this effect might regulate ferroptosis to a great extent.

## 1. Introduction

Morbidity and mortality because of cardiovascular disease during aging are the predominant concerns in both developed and developing countries [1]. Vascular calcification is a hallmark of vascular stiffness and aging [2]. Physiological aging and its impact on vascular calcification are complex and multifactorial, which are governed by a set of mechanisms that influence vascular calcification progression [3]. Furthermore, cardiovascular risk factors, as well as metabolic and degenerative diseases occurring during aging share similar mechanisms that influence vascular aging leading to an increase in vascular calcification [4]. However, by now, very few studies have assessed the specific mechanisms of aging implicated in vascular calcification. Vascular calcification is a prevalent complication that occurs in patients with chronic kidney disease involving mineral metabolism imbalance [5]. Vascular calcification during aging plays a crucial role in cardiovascular disease pathophysiology. The development of vascular calcification is tightly associated with the risk of CVD and all-cause mortality [6], making it a strong independent risk factor for cardiovascular events. The development of vascular calcification is closely linked to mineral dysregulation characterized by the elevation of serum phosphate levels and hypercalcemia. The current hypotheses of vascular calcification development span a wide range of events including inflammation, apoptosis, disruptions to calcium and phosphate homeostasis, matrix vesicle extrusions, osteogenic transformations, extracellular matrix degeneration, and genetic aberrations [6]. Oxidative stress is a state which refers to the imbalance between the oxidative system and antioxidant system in the body. The imbalance between oxidant production and antioxidant defense mechanisms can result from deficient antioxidant capacity caused by the production and distribution of or by an increase in the amount of ROS generated from either endogenous sources or environmental stressors. Excess ROS contributes to the pathological processes related to vascular calcification and the pathogenesis of several vascular calcification-related diseases. Based on the calcification area, vascular calcification can be categorized as external membrane calcification, medial calcification, and valve calcification. However, an accurate mechanism underlying vascular calcification is unclear, making it urgent and meaningful to explore the core causes. The pathological process of vascular calcification shares many similarities with bone formation, and recent studies concluded that it is an initiative course involving phenotypic switching from vascular smooth muscle cells (VSMCs) into osteo/chondroblast-like cells [7]. In response to pathological stimuli, such as high calcium and phosphate levels, oxidative stress, and hypoxia, VSMCs undergo osteogenic differentiation [8]. Under basal conditions, VSMCs in the vessel wall render a contractive phenotype. After the phenotypic switch, osteogenic factors including runt-related transcription factor 2 (RUNX2), Msh homeobox 2 (MSX2), and bone morphogenetic protein 2 (BMP2) will become upregulated with the generation of mineralized extracellular matrix, eventually causing vascular calcification. Senescent vascular smooth muscle cells also play an important role in medial calcification pathophysiology [9]. Some studies have pointed out that aging can increase osteoblastic transition and upregulate Runx2, TNAP(tissue-nonspecific alkaline phosphatase), and type 1 collagen [10]. Also, inorganic phosphate stimulation increases the VSMC senescence during osteochondrogenic dedifferentiation via Sirt-1 inhibition and augmented p53 and histone-3 acetylation [11]. Furthermore, Pi was found to promote senescence in VSMC and vascular calcification in a uremic rat model, which is prevented by phosphate binders [12]. According to N. Giuliani et al., DNA damage response and the senescence-associated secretory phenotype, releasing BMP-2, OPG (osteoprotegerin), IL-6(interleukin-6), and other mediators, which induce the osteogenic phenotype [13]. Although in vitro and in vivo models demonstrated the importance of senescence in VSMC dedifferentiation and vascular calcification, future studies should focus on inhibiting cellular senescence in specific tissues to validate its role in vascular calcification progression.

Ferroptosis is a newly identified type of regulated cell death discovered in recent years that involves metabolic dysfunction [14]. Ferroptosis is usually accompanied by a large amount of iron accumulation and lipid peroxidation during the cell death process. Many studies have shown that ferroptosis is related to the pathology of many diseases, such as cancer, neuron system disease, kidney injury, and blood disease. A recent study revealed that nuclear erythroid 2-related factor 2 (Nrf2) signaling is activated during the regulation of vascular calcification by metformin, suggesting that anti-ferroptosis may be a therapeutic target in calcification [15]. Recent studies have shown that necrosis and apoptosis have unique functions in the progress of vascular development [15], but there is no evidence to show the mechanism of ferroptosis in regulating calcification. The association between atherosclerotic calcification and lipid deposition is well known [16], so it is possible for us to speculate that ferroptosis might play an important role in vascular calcification.

Fra-1, also known as FOSL1, is highly expressed in most solid tumors and is closely related to the proliferation and apoptosis of tumor cells [17]. FOSL1 is a transcription factor, and its own transcriptional activity is regulated at different levels, such as transcriptional regulation and post-translational regulation [18]. FOSL1 can form heterodimer transcription factor complexes with JUN family proteins, thereby regulating the expression of target genes [19]. More recently, studies have reported that FOSL1 regulates several cellular processes that are implicated in inflammation and cell death [20]. However, the potential role of FOSL1 in mediating vascular calcification and ferroptosis has not yet been elucidated.

In this study, we sought to determine how FOSL1 contributes to vascular calcification and aging. We provide evidence that FOSL1 has high expression in vascular calcification both in vivo and in vitro. Inhibiting FOSL1 blocks high calcium and phosphate-induced calcification in VSMCs. Here, we infer that the protective effect of inhibiting FOSL1 might occur through the inhibition of ferroptosis.

## 2. Materials and Methods

### 2.1. Experimental Animals and I/R Models

#### 2.1.1. Animal Experiments

All animal experiments were performed in accordance with the US National Institutes of Health guidelines and were approved by the Institutional Animal Care and Use Committee at Southern Medical University (Guangzhou, China). To avoid the interference of the hormonal changes and exclude the influence of estrogen on vascular calcification, only male mice were used in this study. Adult male C57BL/6 mice aged 6–8 weeks were obtained from Guangzhou Dien Genic Technic (Guangzhou, China) and were allowed free access to standard chow and tap water in temperature and humidity control conditions with a 12 h dark/light cycle. Male C57 mice were randomly divided into the control group (*n* = 12) and the vitD_3_ model group in this study. The vitd3 vascular calcification model was induced by subcutaneous injection of cholecalciferol vitd3 in Cremophor EL and dextrose that was delivered for three consecutive days (5 × 10^5^ IU /kg; [21]. Seven days after the injection, blood samples were collected from the ophthalmic veins and the mice were then sacrificed and animal aortic tissues were excised for further analysis.

#### 2.1.2. Cell Culture

Primary mouse VSMCs were isolated from the thoracic aortas of healthy C57BL/6 mice as previously described [22]. Cells from the first passage were chosen for further experiments and incubated with an osteogenic induction medium to stimulate osteogenic differentiation. VSMCs were grown to confluence and treated with control (1.0 mM Pi/1.8mM Ca) or calcifying media (50 μg/mL ascorbic acid/2.5 mM Pi/2.7 mM Ca) for up to seven days.

### 2.2. Cell Transfection

VSMCs were seeded in 6-well plates and grown in a MEM supplemented with 10% FBS to reach 80% confluence. VSMCs were transfected with siRNA FOSL1 or siRNA NC (RiboBio, Guangzhou, China) with lipofectamine RNA iMAX (Invitrogen, Waltham, MA, USA) in an Opti-MEM (Gibco, Waltham, MA, USA) reduced serum medium. Transfected cells were harvested at indicated time points for further analysis.

### 2.3. Quantitative Real-Time Polymerase Chain Reaction

Total RNA was isolated from VSMCs using a TRIzol (Invitrogen) reagent and reverse-transcribed into cDNA. The polymerase chain reaction primers were as follows: RUNX2 forward: ACCATAACAGTCTTCACAAATCCT, reverse: CAGGCGATCAGAGAACAAACTA; BMP2 forward: GCTTCTTAGACGGACTGCGG, reverse: GCAACACTAGAAGACAGCGGGT; SP7 forward: ATGGCGTCCTCTCTGCTTG, reverse: TGAAAGGTCAGCGTATGGCTT; MSX2 forward: GGAGCACCGTGGATACAGG, reverse: TAGAAGCTGGGATGTGGTGAA; OPG forward: CAGAGAAGCCACGCAAAAGTG, reverse: AGCTGTGTCTCCGTTTTATCCT; FOSL1 forward: ATGTACCGAGACTACGGGGAA, reverse: CTGCTGCTGTCGATGCTTG; P53 forward: TCACAGCGTCTGTTGACATTT, reverse: ACCAAGCTCATTACCCTGACA; SLC7A11 forward: GGCACCGTCATCGGATCAG, reverse: CTCCACAGGCAGACCAGAAAA. Each experiment was repeated at least four times with b-actin as an internal control. Relative gene expression was quantified by the comparative Ct (ΔΔCt; Life Technologies)

### 2.4. Western Blot Analysis

Protein was extracted from VSMCs and vascular tissue using a radioimmunoprecipitation assay lysis buffer, and the bicinchoninic acid method was used for protein quantification. The protein samples were denatured at 95 °C for 5 min with 1× loading buffer. Proteins were separated by SDS-PAGE and transferred to PDVF membranes. After being blocked with 5% BSA for 1 h, blots were incubated with primary antibodies as follows: Anti-RUNX2 (1:2000; Abcam, Cambridge, UK), Anti-MSX2 (1:2000; Santa Cruz Biotechnology, Dallas, TX, USA), Anti-FOSL1 (1:2000; Affinity, Cincinnati, OH, USA), Anti-SLC7A11 (1:5000; Abcam,), Anti-P53 (1:2000; Cell Signaling Technology, Boston, MA, USA), Anti-SP7 (1:2000; Abcam), Anti-OPN (1:2000, Proteintech, Rosemont, IL, USA), or Anti-β-actin (1:2000, Santa Cruz Biotechnology) at 4 °C overnight. After being washed, the membranes were incubated with the HRP-conjugated anti-mouse (1:4000, Cell Signaling Technology Danvers, MA, USA) or anti-rabbit (1:4000, Cell Signaling Technology Danvers, MA, USA) secondary antibodies at room temperature for 1 h before washing. The signal was detected using the AI600 imaging system.

### 2.5. Determination of Calcification

Alizarin Red S (ARS) staining was used to detect VSMC and arterial calcification. For cell staining, cells were fixed in paraformaldehyde for 10 min and then incubated with 2% ARS (pH 4.2) for 30 min at room temperature. To remove excess ARS, cells were washed with ddH2O. For tissue staining, aortas were fixed in 95% ethyl alcohol at 4 °C for 24 h, then washed in 0.5% KOH and stained with 0.0016% alizarin red for 12 h. Samples were stored in 50% glycerin until use. Photos were taken by a stereomicroscope and calcium content was measured using a calcium colorimetric assay kit (Sigma-Aldrich, St. Louis, MO, USA) per the manufacturer’s instructions.

### 2.6. Arterial Ring Organ Culture

Thoracic aortas were isolated from 6–8-week-old male C57BL/6 mice and then incubated in calcifying medium for seven days. Arterial rings were grown to confluence and treated with control (1.0 mM Pi/1.8 mM Ca) or calcifying media (50 μg/mL ascorbic acid/2.5 mM Pi/2.7 mM Ca) for up to seven days.

### 2.7. GSH Assay

The GSH contents in cells were quantified using a commercial GSH determination kit (Promega, Madison, WI, USA). Cells were plated in a 96-well luminometer-compatible plate and allowed to grow overnight at 37 °C in a 5% CO_2_ culture incubator. At the end of the treatment, the growth medium was removed and replaced with a test compound medium. Detection was performed by measuring the absorbance value at a wavelength of 562 nm using a microplate reader (Bio-Rad 680, Hercules, CA, USA). The GSH level was calculated by subtracting the amount of GSSG from the total GSH.

### 2.8. ROS Assay

The intracellular ROS content was measured with the fluorescent probe DCFH- DA (Solarbio, China), which itself is nonfluorescent. After freely passing through the cell membrane, Tt is hydrolyzed to impenetrable DCFH and remains inside the cells. Intracellular ROS oxidizes DCFH to fluorescent DCF, which indicates the intracellular ROS levels according to the fluorescence intensity. VSMCs with five different treatments were incubated with serum-free DMEM with a DCFH-DA final concentration of 10 uM for 30 min at 37 °C. Next, we washed the VSMC-DCFH-DA mixture with serum-free DMEM three times and resuspended it in 1× PBS solution. The prepared VSMC samples were analyzed using flow cytometry (Life Attune NxT, Thermo Fisher Scientific, Waltham, MA, USA). The results were analyzed with Flowjo 10 software.

### 2.9. Statistical Analysis

All results are expressed as the mean ± SEM. Statistical analysis was performed using SPSS statistical software. Independent student’s *t*-tests were used to test the differences between the two groups, and comparisons among multiple groups were performed by one-way ANOVA. Differences were accepted as statistically significant at *p* < 0.05.

## 3. Results

### 3.1. FOSL1 Expression Is Induced after vitD3 Treatment In Vivo

**Figure 1:**
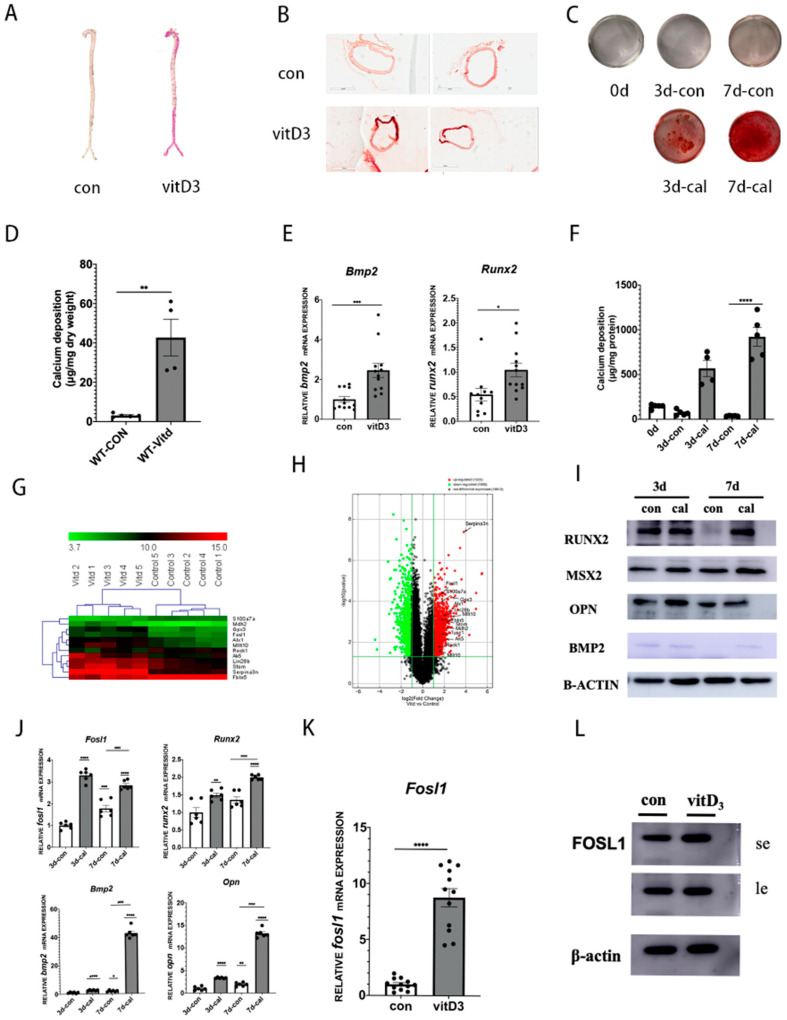
FOSL1 is up-regulated in vascular calcification both in vivo and in vitro. (**A**) Representative ARS staining of whole aortic arteries isolated from C57 mice (original magnification scale bars =), *n* = 3 per group. (**B**) Mouse arteries were isolated from vitD3 animal models, mineral deposition in the aortic section was detected by ARS staining. (**C**) Calcium content of arteries was measured by a calcium assay kit, *n* = 5 independent experiments. (**D**) Calcium deposition in aortas was detected by ARS staining.; *n* = 4 per group. (**E**) mRNA expression levels of osteogenesis genes in animal aortas were analyzed by RT-qPCR, *n* = 12 per group. (**F**) Mouse vascular smooth muscle cells (VSMCs) were incubated in the presence of calcifying medium for seven days. Mineral deposition in VSMCs was detected by ARS staining, *n* = 5 per group. (**G**) Heatmap of differentially expressed genes, *n* = 5. (**H**) Volcano plot showing FOSL1 as one of the most upregulated genes in microarray analysis: log 2-fold change versus log 2-fold regulation, *n* = 50 per group. (**I**) Western blot analysis of RUNX2, MSX2, OPN, and BMP2 protein expression, *n* = 3 per group. (**J**) Quantitative real-time polymerase chain reaction analysis of FOSL1, BMP2, RUNX2, and OPN mRNA expression in mouse VSMCs, *n* = 5 per group. (**K**) FOSL1 mRNA expression in animal arteries, *n* = 12 per group. (**L**) FOSL1 protein expression in mouse arteries, *n* = 3 per group. Statistical analysis was performed using one-way ANOVA (Tukey honestly significant difference post hoc test) * *p* < 0.05, ** *p* < 0.01, *** *p* < 0.001, **** *p* < 0.0001.

To identify genes involved in vascular calcification, we first established a vitd3 mouse model as previously described [23] (Figure 1A). ARS staining showed that the calcification of the aorta is well developed a week after vitd3 treatment, with no obvious change in the control group. Both RT-qPCR and western blot analysis revealed that the osteogenic genes BMP2 and RUNX2 were significantly increased in the vitD3-treated group compared with the control group (Figure 1E). Consistently, paraffin sections showed that the aortas of vitD3 group mice had serious mineral deposits (Figure 1B). We then sent samples of calcified aortas for silicon screening, and we found many differentially expressed genes between the two groups, with FOSL1 being one of the most up-regulated genes (Figure 1G,H).

### 3.2. Expression of FOSL1 Was Increased in Vascular Calcification Both In Vivo and In Vitro

To verify the expression of FOSL1, we utilized primary-culture VSMCs. Methods for isolating mouse primary cells were as previously described [24]. VSMCs were treated with a high concentration of Pi and Ca2^+^, which can efficiently recapitulate vascular calcification as shown in our previous study [25]. ARS showed that VSMCs treated with high amounts of Pi and Ca2^+^ markedly promoted calcification at day seven (Figure 1C). Consistently, calcium content assays revealed that high Pi- and Ca2^+^-treated cells showed increased calcium levels compared with the control group (Figure 1F). Also, high Pi and Ca2^+^ treatment promoted osteogenic differentiation of VSMCs, as indicated by the increased expression of BMP2, RUNX2, MSX2, and OPN, both at the mRNA and protein level. Next, we confirmed that the expression of FOSL1 was also upregulated at both the mRNA and protein level (Figure 1J,I) and the quantitative analysis of the western blotting (Figure S1). We further analyzed the expression of FOSL1 in vivo by extracting mRNA and protein from vitD3 mice. Consistent with previous results, FOSL1 was significantly upregulated in the vitd3 group compared with the control group (Figure 1K,L) and the quantitative analysis of the western blotting (Figure S2).

### 3.3. Knockdown of FOSL1 Attenuated VSMC Calcification

**Figure 2:**
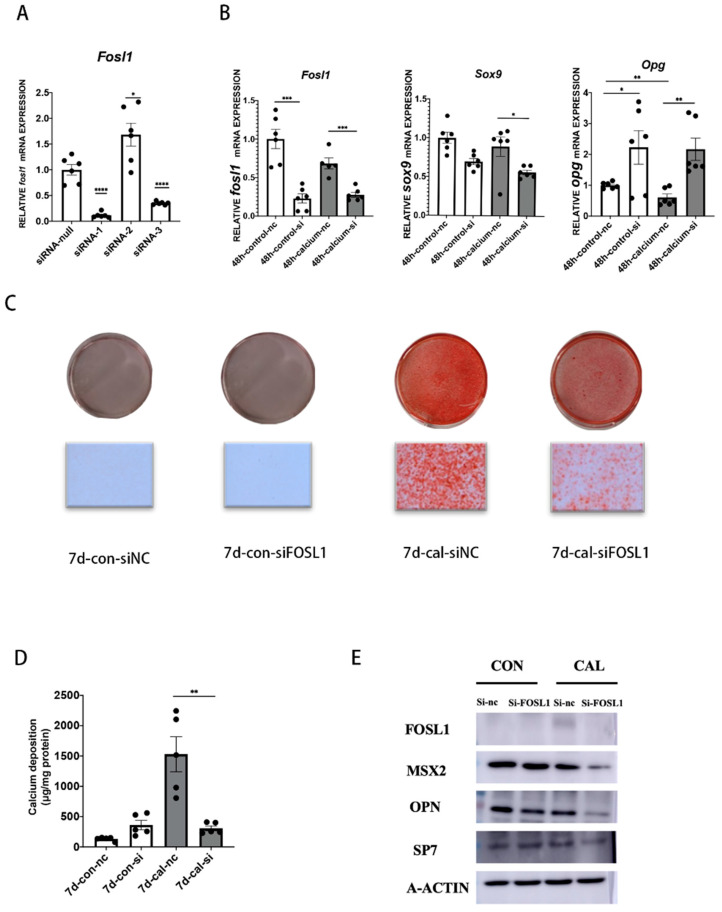
Effect of FOSL1 on vascular calcification of VSMCs. (**A**) mRNA expression of FOSL1 was analyzed after being knocked down by three siRNAs for 48 h, *n* = 6 per group. (**B**) RT-qPCR was used to detect the expression of FOSL1, SOX9, and OPG, *n* = 6 per group. (**C**) Mouse VSMCs were transfected with siFOSL1 and siNC under calcium medium for seven days. ARS staining was used to detect mineral deposition. (**D**) In accordance with C, calcium content was measured by a calcium content assay kit, *n* = 5 per group. (**E**) Western blot analysis of FOSL1, MSX2, OPN, and SP7, *n* = 3 per group. Statistical analysis was performed using one-way ANOVA (Tukey honestly significant difference post hoc test) * *p* < 0.05, ** *p* < 0.01, *** *p* < 0.001, **** *p* < 0.0001.

Given the fact that the FOSL1 gene is significantly amplified and overexpressed in calcification areas, we next investigated whether the alteration of the transcriptional master regulator FOSL1 affects VSMC calcification. As FOSL1 was identified as one of the most effective reduction targets in protecting vascular calcification in the tissue silicon screen, we next asked whether FOSL1 can actually have a function in the process of vascular calcification. Using specific FOSL1-targeting siRNAs, we first sought to validate the most effective one which can reduce the expression of FOSL1 at both the mRNA and protein level in primary VSMCs (Figure 2A). FOSL1 deficiency noticeably alleviated VSMC calcification, and ARS staining of aortas and calcium content showed a decrease in VSMC calcification in the siFOSL1-treated group (Figure 2C,D). Moreover, qPCR and western blotting showed reduction of FOSL1 can also decrease the expression of the osteogenic genes MSX2, SP7, and OPN (Figure 2B,E), and the quantitative analysis of the western blotting showed the same trend.(Figure S3). These results together demonstrate that FOSL1 has a function in the regulation of vascular calcification.

### 3.4. Ferroptosis Can Aggravate VSMC Calcification and ROS Generation

**Figure 3:**
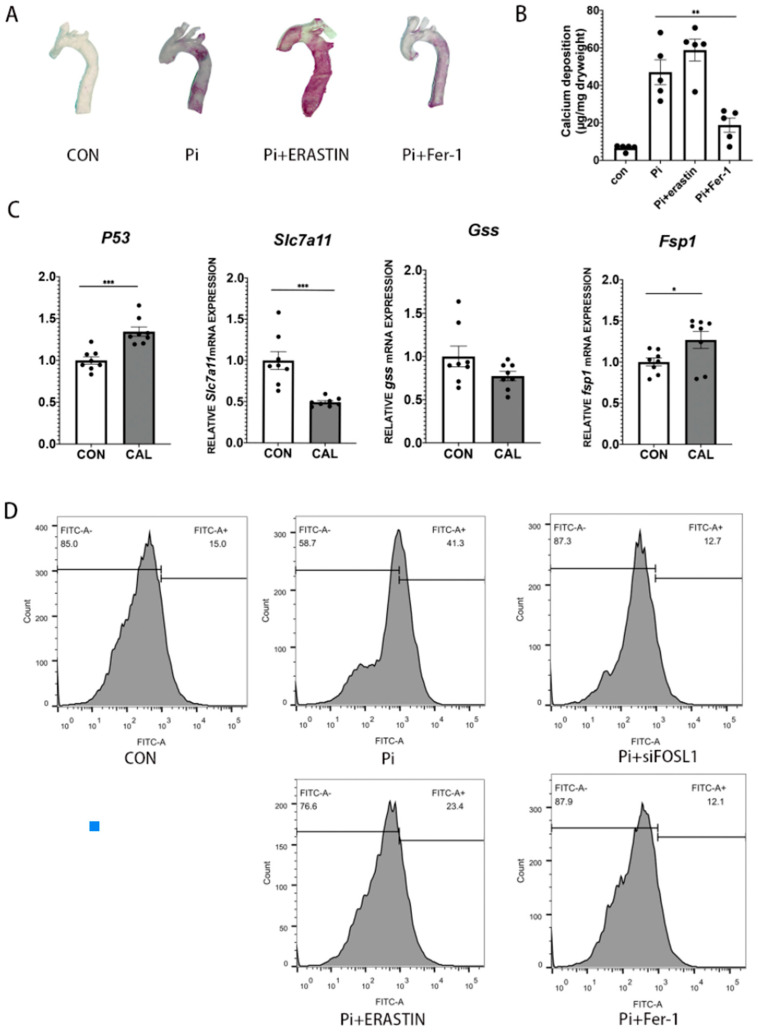
Ferroptosis can aggregate vascular calcification and cellular ROS generation. (**A**) Mineral deposition in mouse aortic rings incubated with erastin (5 mM) or fer-1 (5 mM) in the presence of calcifying medium for five days, *n* = 4. (**B**) Calcium content was measured as described in the Section 2. (**C**) P53, SLC7A11, GSS, and FSP1 mRNA expression were determined by western blot, *n* = 8 per group. (**D**) ROS generation with Pi, Pi+siFOSL1, Pi+erastin, and Pi+Fer-1 treatments compared with normal control, *n* = 3 per group. Statistical analysis was performed using one-way ANOVA (Tukey honestly significant difference post hoc test) * *p* < 0.05, ** *p* < 0.01, *** *p* < 0.001.

Many studies have pointed out that apoptosis has an important role in regular mineral balance, which is the core mechanism in VSMC calcification [26]. However, there is no specific explanation for the role of ferroptosis in the process of VSMC calcification. As shown in Figure 3A,B, high Pi concentration can induce serious calcification in the aortic ring, which was more aggregate in the presence of the ferroptosis-inducing drug erastin, and the calcification can be relieved by Ferrostatin-1 (fer-1), a drug to protect cells from ferroptosis [27]. These data show that ferroptosis can aggravate VSMC calcification.

### 3.5. Inhibition of FOSL1 Can Attenuate VSMC Calcification and ROS Generation through Upregulation of SLC7A11 and Inhibiting Ferroptosis

Next, we investigated whether the protective effect of inhibiting FOSL1 in VSMC calcification is attained through regulating ferroptosis.

We harvested mouse aortic VSMCs and assigned five treatments: normal control, Pi+calcium, Pi+calcium+siFOSL1, Pi+calcium+erastin, and Pi+calcium+Fer-1. The ROS content calculated by fluorescence intensity significantly increased under high Pi and calcium treatments compared with the normal control. In addition, the ROS content also increased after adding erastin to the Pi treatment. These results indicated that high Pi and calcium conditions promoted oxidative stress in VSMCs. Conversely, ROS generation significantly decreased after knocking down FOSL1 and Fer-1 treatment. These results suggested that FOSL1 is closely related to intracellular oxidative stress in VSMCs under calcification conditions. Moreover, ferroptosis might aggravate vascular calcification by upregulating intracellular ROS production.

**Figure 4:**
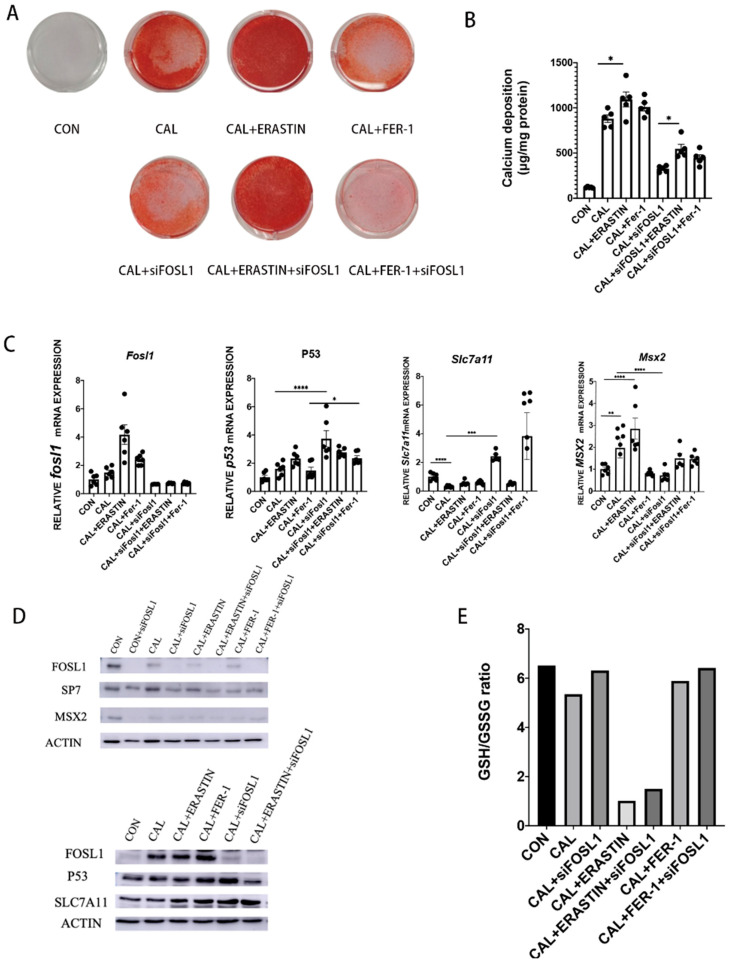
Inhibition of FOSL1 can attenuate VSMC calcification through upregulation of SLC7A11 and inhibition of ferroptosis. (**A**) Mineral deposition in VSMCs was detected by ARS staining and (**B**) calcium content assay; *n* = 5 per group. (**C**) P53, SLC7A11, GSS, and FSP1 mRNA expression were determined by RT-qPCR; *n* = 6 per group. (**D**) Western blot analysis of FOSL1, P53, SLC7A11, SP7, and MSX2 protein expression; *n* = 3 per group. (**E**) Cellular GSH levels were measured as described in the Section 2; *n* = 3 per group. Statistical analysis was performed using one-way ANOVA (Tukey honestly significant difference post hoc test) * *p* < 0.05, ** *p* < 0.01, *** *p* < 0.001, **** *p* < 0.0001.

Ferroptosis has been reported to be involved in many pathological processes including cancer development, traumatic brain injury, and coronary artery disease, and SLC7A11 is a critical key factor in the processing of ferroptosis. As shown in Figure 3C, the level of SLC7A11 was significantly reduced in the calcifying group, and inhibiting FOSL1 can enhance the expression of SLC7A11 (Figure 4C,D). In addition, we measured the GSH levels of VSMCs. GSH level is an indicator of redox reactions in cells, which is the main convert in the process of ferroptosis. As shown in Figure 4E, GSH levels were significantly upregulated when we knocked down FOSL1. So far, our data suggested the protection of reducing FOSL1 in VSMC calcification was partly through inhibiting ferroptosis.

## 4. Discussion

Cellular senescence is defined as an irreversible loss of proliferation potential. Cellular senescence contributes to vascular aging and vascular calcification. Characterized as a permanent cell cycle arrest of mitotic cells, which does not respond to various stimuli and undergo proinflammatory and proatherosclerotic phenotypic changes. Vascular calcification is a very complex pathological process that is characterized by reduced vascular wall elasticity and compliance [28]. It is an independent risk factor and an established predictor for cardiovascular disease. Recent studies have considered it an active cell-regulated process, and osteogenic differentiation of vascular smooth muscle cells plays a vital role in the development of vascular calcification. The imbalance of ROS generation systems and antioxidant systems can cause oxidative stress, which is closely related to key events in vascular calcification progress, such as phosphate imbalance, VSMC differentiation, inflammatory reaction, DNA damage, and extracellular matrix remodeling. Therefore, understanding and elucidating the relationship between ROS generation and vascular calcification might emphasize the role of antioxidants in treating vascular calcification. Our study’s main aim is to find a therapeutic target for vascular calcification by exploring the regulatory mechanism during the progression of vascular calcification.

In this study, we identified a transcriptional factor, FOSL1, that is involved in the processes of high-phosphate and calcium-induced calcification in VSMCs. Additionally, for the first time, we demonstrated the contribution of ferroptosis in the regulation of vascular calcification. We found that FOSL1 inhibition attenuates vascular calcification and decreases the expression of osteogenesis genes. In addition, we observed that reducing FOSL1 also has an effect on SLC7A11 and P53, which are two major genes in regulating ferroptosis. Our findings provide new insights into the mechanisms of the protection caused by inhibiting FOSL1 in vascular calcification, which is not only through traditional osteogenic differentiation but also through the regulation of ferroptosis.

FOSL1 has been shown to be involved in a wide range of cellular events, such as cell proliferation, cell growth, and apoptosis [17]. FOSL1 is a transcription factor belonging to the AP-1 family, which consists of many homodimers and heterodimers [29]. FOSL1 is one of the most important subunits of the AP-1 complex. Many clinical studies have shown that FOSL1 has considerably high expression in many solid tumors and is associated with poor prognosis in patients [30]. In this study, we found that FOSL1 expression is strongly up-regulated in vascular smooth muscle cells and aortas during vascular calcification. FOSL1 has also been shown to regulate cartilage development. Until now, it has not been clear whether FOSL1 contributes to the development of vascular calcification, but we show that inhibiting FOSL1 by siRNA attenuates the calcification of VSMCs. For the first time, we show that ferroptosis is involved in FOSL1-mediated VSMC calcification. These finding could provide new insights into the pathogenesis of vascular calcification.

Previous studies have demonstrated that FOSL1 is essential for chondrogenesis and bone mineralization [31]. One study revealed that FOSL1 can directly activate P21 and P16 signaling to promote vascular senescence [32], and vascular aging is one of the principal risk factors owing to the high mortality of cardiovascular disease. In the endothelial domain, Fosl1^−/−^ embryos develop vascular defects in extraembryonic tissues and die [32]. Embryonic stem cells lacking FOSL1 differentiate into endothelial cells but do not form primitive capillaries or tube-like structures, and FOSL1 is required for human umbilical vein endothelial cell (HUVEC) assembly into vessels [33]. FOSL1 has also been reported to modulate angiogenesis and β3 integrin and endothelial cell adhesion [33]. FOSL1 can also act as a transcriptional regulator of angiogenesis-related genes [34]. The potential function of FOSL1 in vascularity is highly probable, and the mechanism of FOSL1 regulating vascular angiogenesis and calcification is worth exploring.

As previously mentioned, several studies have found that multiple forms of cell death may play a role in the pathogenesis of vascular calcification, and preventing VSMC death can be an effective strategy for vessel protection [28]. Lipid peroxidation and inflammation are two major risk factors contributing to atherosclerosis [16]. One study found that atherosclerosis-prone apolipoprotein E-deficient (ApoE^−/−^) mice induced atherosclerosis in the presence or absence of a commonly used ferroptosis inhibitor fer-1. Inhibition of ferroptosis can significantly attenuate atherosclerotic lesions and partially inhibit iron accumulation and lipid peroxidation, and reverse the enhanced expression of SLC7A11 and GPX4 compared with the wide type [35]. Also, atherosclerosis has been known to cause ischemia reperfusion (IR) with severe cell damage and death [36]. In a rat model of IR with diabetes, Li et al. found that inhibition of ferroptosis by fer-1 attenuated ER stress and alleviated myocardial damage, whereas promoting ferroptosis by erastin aggravated cell damage [14]. In summary, accumulating evidence appears to demonstrate that ferroptosis is involved in cardiovascular disease, so the role of ferroptosis is worthy of exploration.

Although this study presents significant novel findings, it still has several limitations. First, the direct regulated relationship between FOSL1 and SLC7A11 needs to be further validated, and the specific signaling needs to be confirmed. Second, as a transcription factor, we speculate that there is a strong possibility that FOSL1 directly regulates SLC7A11, and further experiments are needed to prove this theory. Third, to better illustrate the relationship between ferroptosis and vascular calcification, clinical patient blood samples could be used to provide this evidence. Finally, detecting ferroptosis levels in patients with serious vascular calcification could show the potential relationship between the disease and pathology process in a more intuitive way.

## 5. Conclusions

In conclusion, this study advances a better understanding of vascular calcification during aging by providing a novel mechanistic link between ferroptosis and vascular calcification. We observed a significant protective effect when we inhibited the expression of FOSL1 during vascular calcification, which may occur through regulating ferroptosis. We also demonstrated for the first time that inhibition of FOSL1 can attenuate vascular calcification and osteogenic differentiation of VSMCs in the context of the P53-SLC7A11 axis, though the next step is providing evidence of the direct regulatory relationship. Our data suggest that reducing the levels of FOSL1 could become a potential treatment strategy for vascular calcification. 

## Data Availability

Not applicable.

## Supplementary Materials

The following supporting information can be downloaded at: https://www.mdpi.com/article/10.3390/biomedicines11020635/s1, Figure S1: Semi-quantitative analysis of the western blotting data presented in Figure 1I; Figure S2: Semi-quantitative analysis of the western blotting data presented in Figure 1L; Figure S3: Semi-quantitative analysis of the western blotting data presented in Figure 2E; Figure S4: quantitative analysis of the ROS generation data presented in Figure 3D; Figure S5: Semi-quantitative analysis of the western blotting data presented in Figure 4D; Figure S6: Semi-quantitative analysis of the western blotting data presented in Figure 4D.
