# Supplementary material for: Influence of FOSL1 Inhibition on Vascular Calcification and ROS Generation through Ferroptosis via P53-SLC7A11 Axis
Source: Biomedicines. 2023 Feb 20;11(2):635. doi: 10.3390/biomedicines11020635 (PMC9953509; doi:10.3390/biomedicines11020635)
Supplement: Supplementary file 1 [file biomedicines-11-00635-s001.zip › biomedicines-2191413-supplementary materials.pdf]

S1

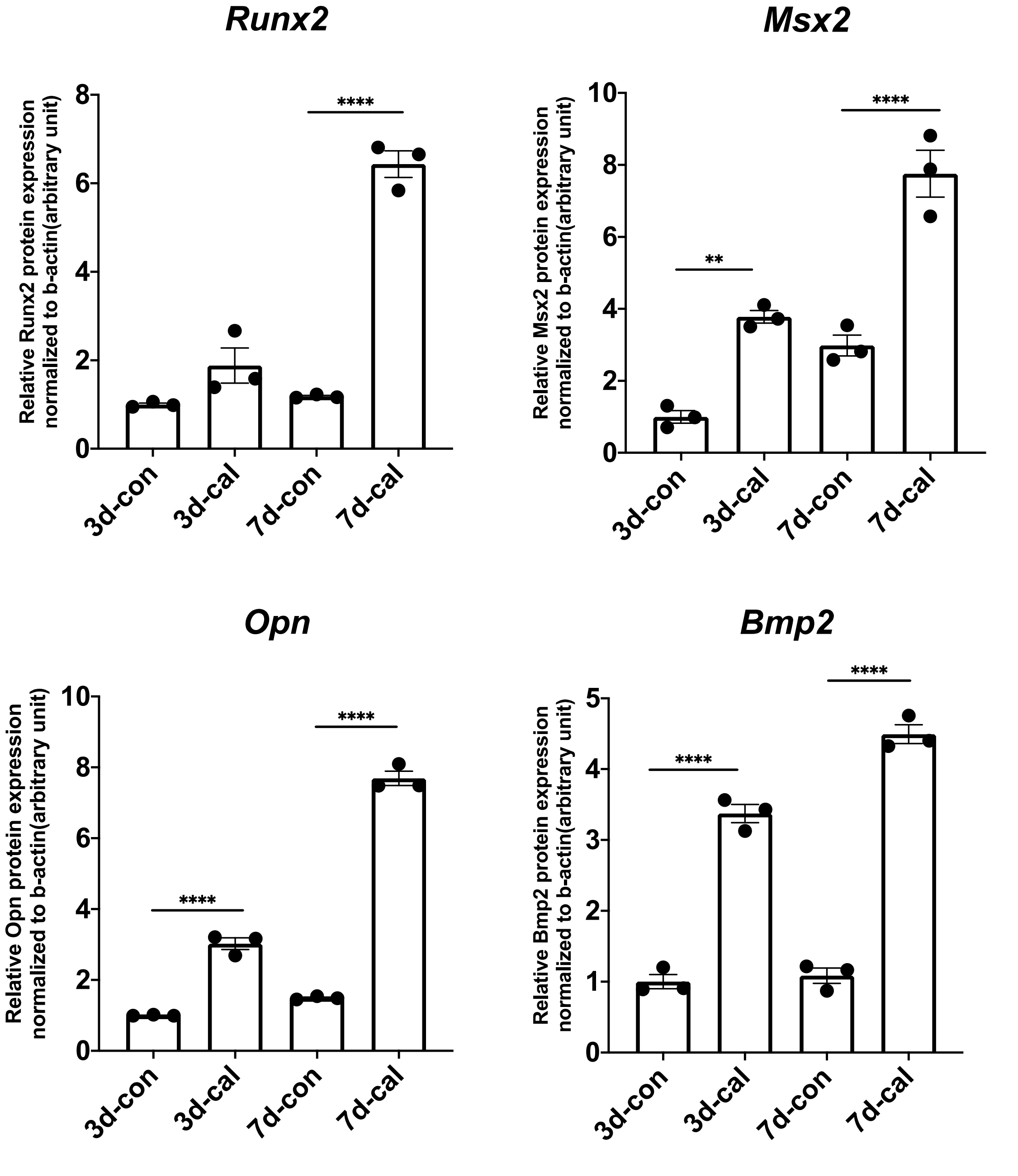

Figure S1.Semi-quantitative analysis of the western blotting data presented in Figure 1I. n=3.Data are presented as mean ± SEM, and statistical significance was analyzed by a two-tailed unpaired Student’s t-test.

S2

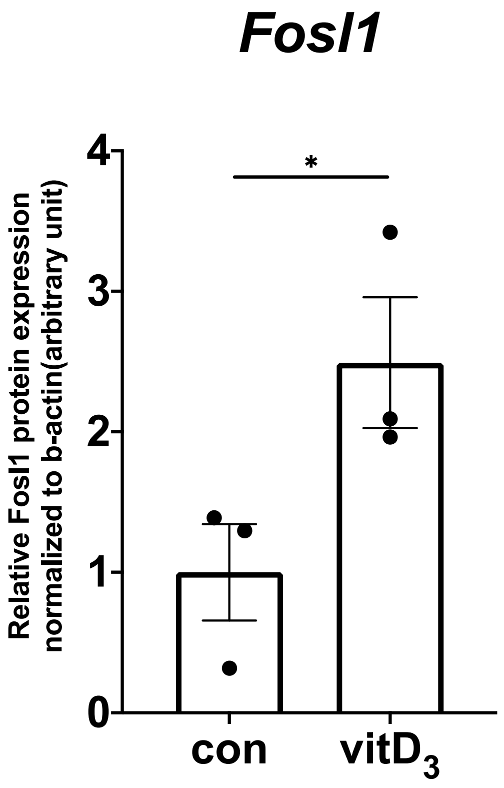

Figure S2.Semi-quantitative analysis of the western blotting data presented in Figure 1L. n=3.Data are presented as mean ± SEM, and statistical significance was analyzed by a two-tailed unpaired Student's t-test.

S3

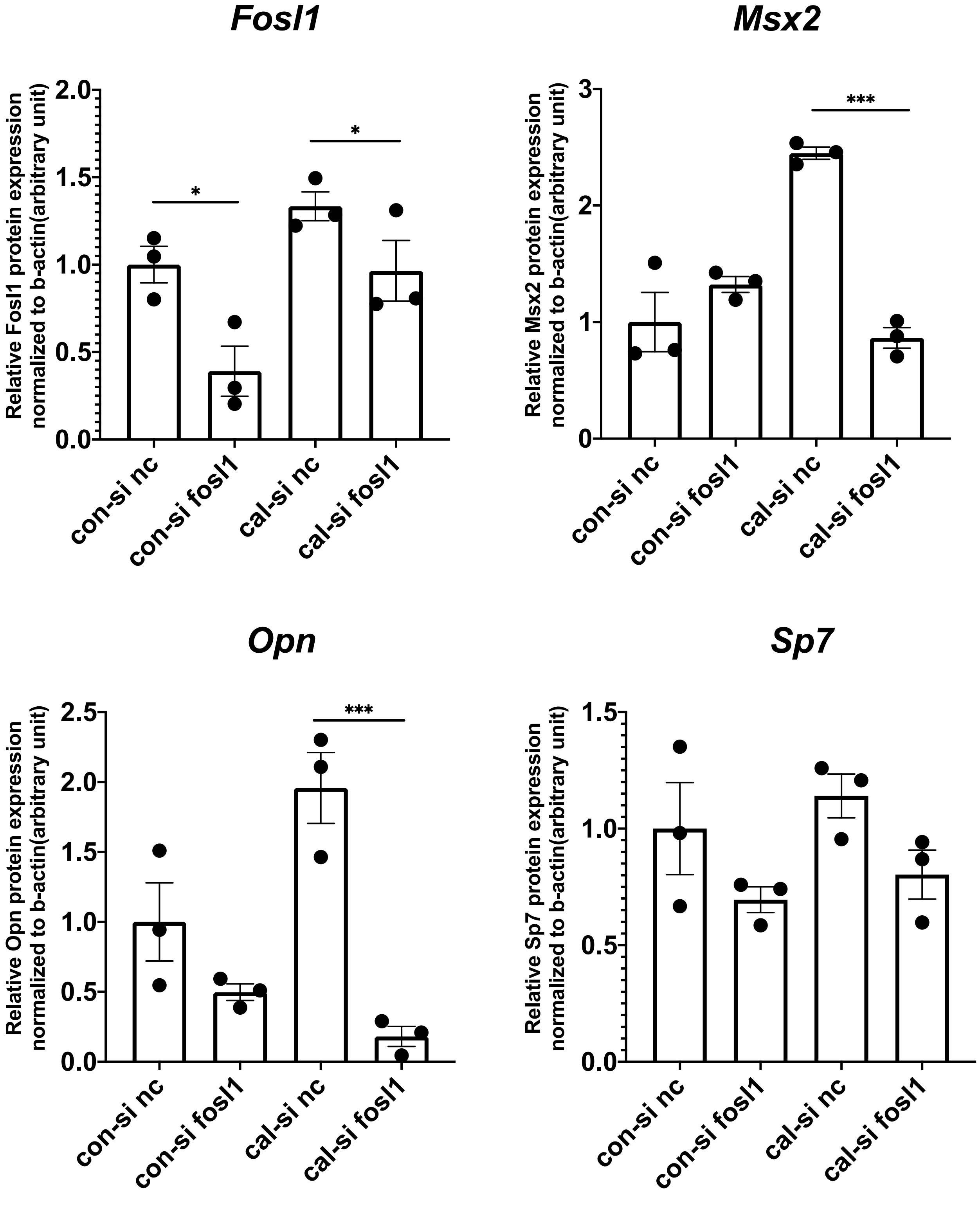

Figure S3.Semi-quantitative analysis of the western blotting data presented in Figure 2E. n=3.Data are presented as mean ±

SEM, and statistical significance was analyzed by a two-tailed unpaired Student’s t-test.

S4

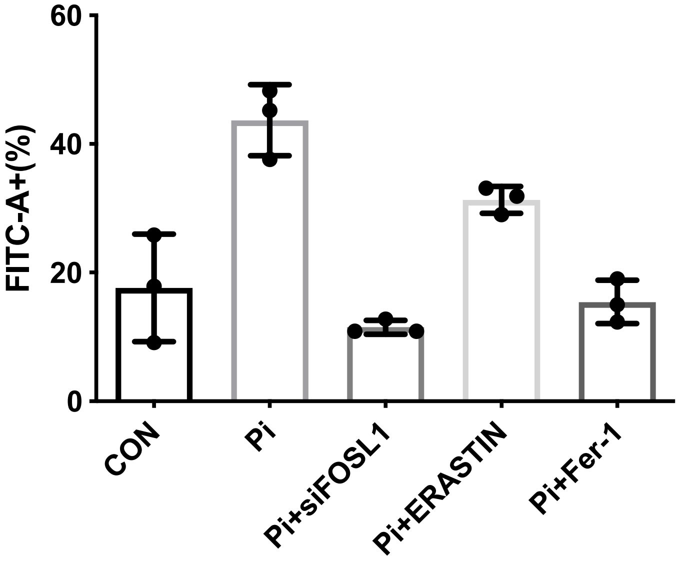

Figure S4. quantitative analysis of the ROS generation data presented in Figure 3D. n=3.Data are presented as mean ± SEM, and statistical significance was analyzed by a two-tailed unpaired Student’s t-test.

S5

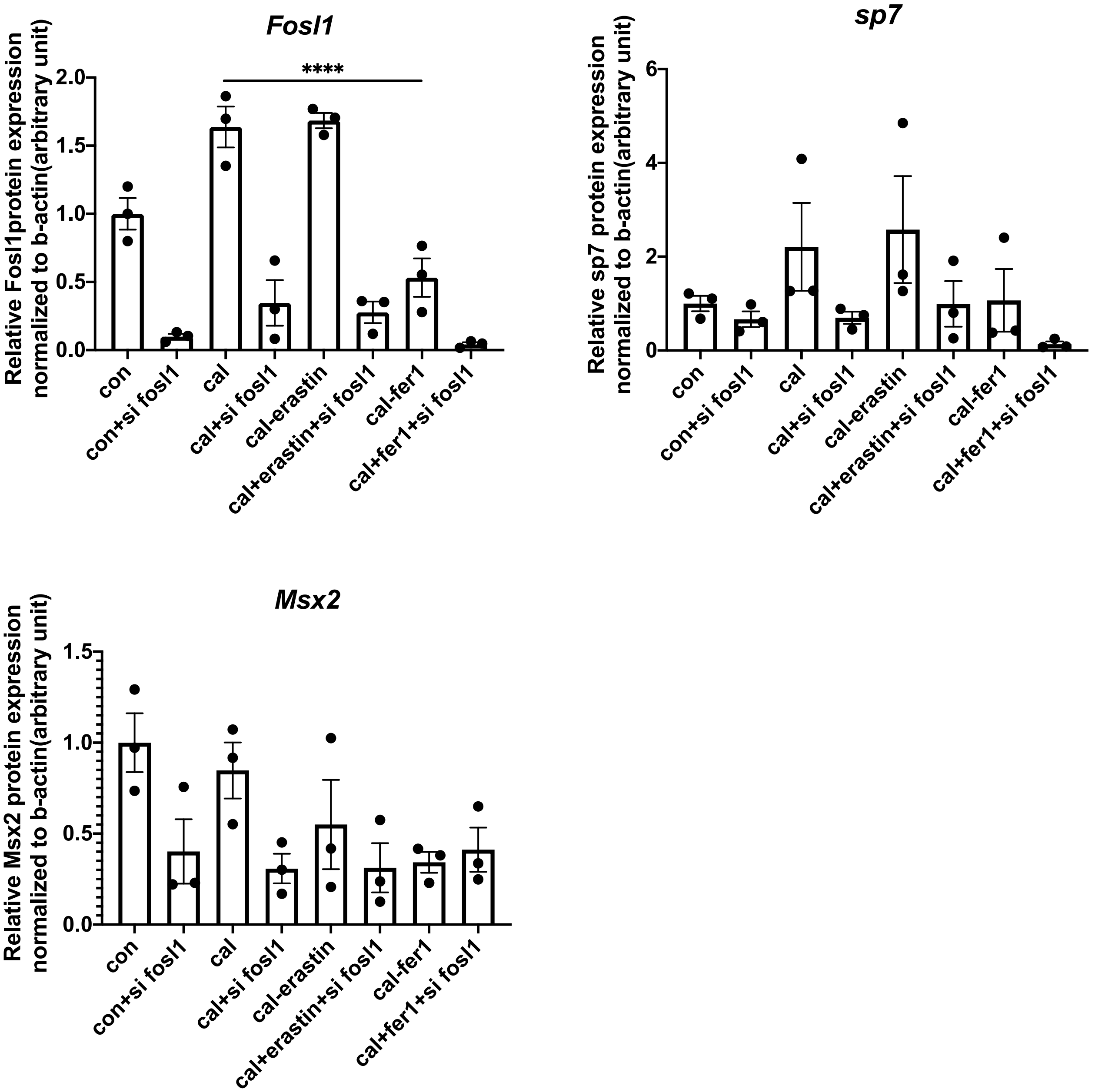

Figure S5.Semi-quantitative analysis of the western blotting data presented in Figure 4D. n=3.Data are presented as mean ± SEM, and statistical significance was analyzed by a two-tailed unpaired Student’s t-test.

S6

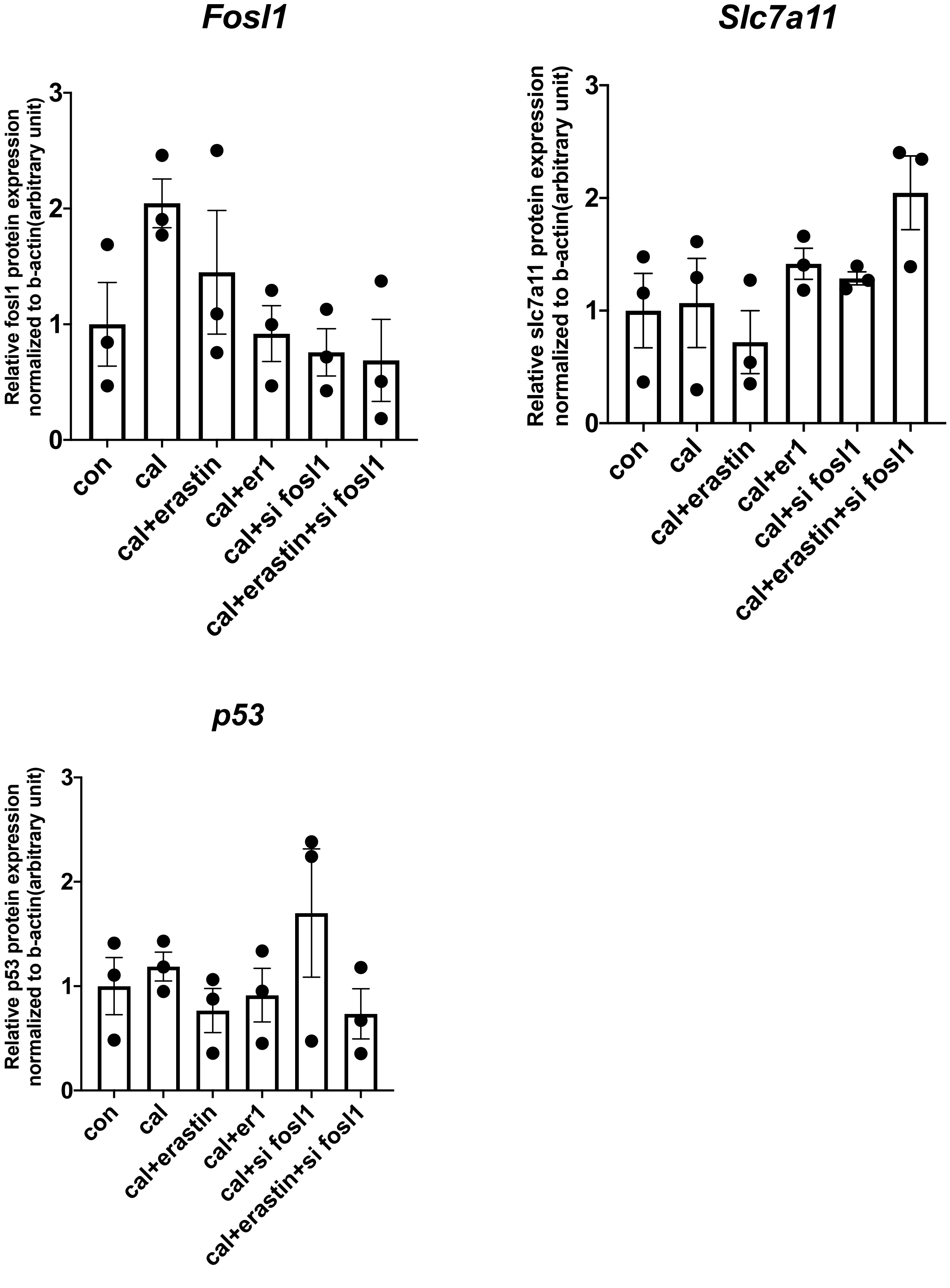

Figure S6.Semi-quantitative analysis of the western blotting data presented in Figure 4D. n=3.Data are presented as mean ±

SEM, and statistical significance was analyzed by a two-tailed unpaired Student’s t-test.
